# Study on mechanical properties of aeolian sand solidified by enzyme induced carbonate precipitation combined with fiber reinforced

**DOI:** 10.1038/s41598-025-18375-2

**Published:** 2025-09-25

**Authors:** Jia Liu, Jianxiang Qin, Gang Li, Jing Qu

**Affiliations:** Shaanxi Key Laboratory of Safety and Durability of Concrete Structures, Xijing University, Xi’an, Shaanxi 710123 China

**Keywords:** Aeolian sand, EICP, Fiber reinforcement, Unconfined compression strength, Poly model, Biomaterials, Civil engineering

## Abstract

Aeolian sand is a problematic soil formed by the transfer, transition, and deposition of rock particles in desert areas, which can easily cause land desertification. Traditional sand treatment methods are generally contrary to the strategy of green and low carbon, whereas enzyme-induced calcium carbonate precipitation (EICP) is an efficient, green, and durable solidification method, and coupled with fiber reinforcement, can significantly improve the sand strength and reduce brittle fracture. This paper investigated the mechanical characteristics of aeolian sand solidified by enzyme-induced calcium carbonate precipitation coupled with fiber reinforcement by conducting permeability and unconfined compression strength (UCS) tests. The test results indicated that the optimal solidified conditions for the EICP were that the dry density, bonding times, standing time, and enzyme cement ratio were 1.6 g/cm^3^, 5 times, 5 days, and 1:1, respectively. Under these conditions, the UCS reached its maximum of 392.52 kPa. When the fiber length and fiber content of basalt fiber were 6 mm and 0.75%, and that of wool fiber were 9 mm and 0.75%, respectively, the optimal reinforcement conditions were achieved, yielding maximum UCS values of 849.65 kPa and 885.31 kPa, respectively. Based on the test results, the solidified mechanism was revealed, involving pore filling and particle cementation by EICP products and the formation of a three-dimensional reinforcement network by fibers, synergistically enhancing strength and transforming brittle sand into a ductile solidified body. A Poly model considering fiber length, fiber content, and UCS was established and validated, showing excellent agreement with experimental data. The research results can offer a valuable guideline for the treatment of aeolian sand in desert areas.

## Introduction

Desertification is one of the most serious ecological and environmental problems in the world^1^. According to statistics, about 40% of the world’s land area and more than 30% of the population are deeply affected by desertification. In China, the area of desertified land accounts for 17.58% of the total land^2^. Although the area of desertified land is decreasing every year, the overall situation is still quite serious. Aeolian sand is a problematic soil formed by the transfer, migration, and deposition of soil particles in desert areas, characterized by loose structure, poor gradation, no cohesion, low water retention, and low bearing capacity. It is very easy to loosen and deform under external forces, leading to construction challenges. Therefore, it is very necessary to stabilize it.

Traditional aeolian sand reinforcement methods include physical and chemical sand fixation^3,4^but most of them have a significant negative influence on the environment. In response to the Sustainable Development Goals, eco-friendly soil stabilization technologies are urgently needed. At present, there are many environmentally friendly modification strategies used to enhance the fracture toughness of concrete composite materials^5^. Among these modification strategies, EICP stands out as an efficient, eco-friendly soil stabilization technology, which researchers from diverse fields have shown extensive interest in it^6–11^. Extensive studies demonstrate its effectiveness in enhancing the mechanical properties of aeolian sand. Arab et al.^12^ detected that the UCS of aeolian sand was significantly improved after one cycle of treatment with jellyfish powder as an enzyme source. Wu et al.^13^ detected that the UCS rose when the activity of urease rose from 2.95 U/mL to 5.39 U/mL and the activity of binder rose from 0.25 M to 0.75 M. Li et al.^14^ found that the aeolian sand solidified by EICP exhibited strain softening. The peak strength increased with the increase of initial dry density, bonding times, standing time, and confining pressure through consolidation undrained triaxial tests. Zomorodian et al.^15^ studied the amelioration of the erosion resistance of the sample surface mediated by 10 different EICP treatment schemes, and found that all 10 EICP treatment schemes significantly improved the erosion resistance. Yuan et al.^16^ conducted permeability tests and UCS tests, and found that with the increase of initial dry density, bonding amount, and standing time, the permeability coefficient of the solidified sand decreased and the UCS increased. Gitanjali et al.^17^ used EICP technology to increase the intensity, stiffness, and resistance to loess liquefaction by blocking pores and combining soil particles with CaCO_3_. Despite these advances, EICP faces inherent constraints, particularly the lack of nucleation sites, which affects the soil stabilization effect^18,19^.

In order to overcome these limitations, fiber-reinforced EICP or MICP systems have gained significant attention^20–22^. Cui et al.^23^ used EICP combined with polypropylene fiber technology to solidify aeolian sand, and found that the method can improve the UCS of solidified sand. Alotaibi et al.^24^ explored the feasibility of using macroscopic synthetic fibers to improve the mechanical properties of EICP-reinforced soil and found that adding fibers to EICP-treated soil can increase the residual compressive strength and failure strain of the soil. Yao et al.^25^ investigated the mechanical properties of MICP-cured loose sand using wool fibers as reinforcement materials, and found that wool fibers significantly improved the strength of MICP-treated sand through crack bridging and energy absorption. Shan et al.^26^ analyzed the effect of different polyester fiber and hemp fiber contents on the dynamic characteristics of the MICP–fiber combined reinforced calcareous sand under small-strain conditions, and found that the maximum dynamic shear modulus of the small-strain of MICP fiber composite reinforced lime sand showed an upward trend with the increase of fiber content. Han et al.^27^ found that the introduction of DFMF significantly enhanced the ductility of sand treated with MICP.

In summary, significant achievements were made in the research of combining EICP or MICP with a single type of fiber for soil reinforcement. The research on the synergistic solidification of aeolian sand by EICP and dual fiber systems formed by different fiber types is still limited, and there is a lack of quantitative models for the relationship between fiber parameters and strength. Therefore, the study took the aeolian sand in the Maowusu Desert in Yulin City, Shaanxi Province as the research object. Based on permeability tests and unconfined compressive strength tests, the optimal solidification conditions for EICP solidified aeolian sand were obtained. Based on the optimal solidification conditions, an unconfined compressive strength test was conducted, and the effects of fiber type, fiber length, and fiber content on the unconfined compressive strength of the sample were analyzed under the conditions of EICP combined with basalt fiber or wool fiber to solidify aeolian sand. The optimal reinforcement conditions were ultimately obtained, and the mechanism of EICP combined with fiber reinforcement in solidifying aeolian sand was analyzed. A Poly model representing the relationship between fiber length, fiber content, and UCS was eventually established and validated, with further discussion on its engineering applications and limitations. The research results can offer a valuable guideline for the engineering application of aeolian sand.

## Materials and methods

### Test materials

**Fig. 1:**
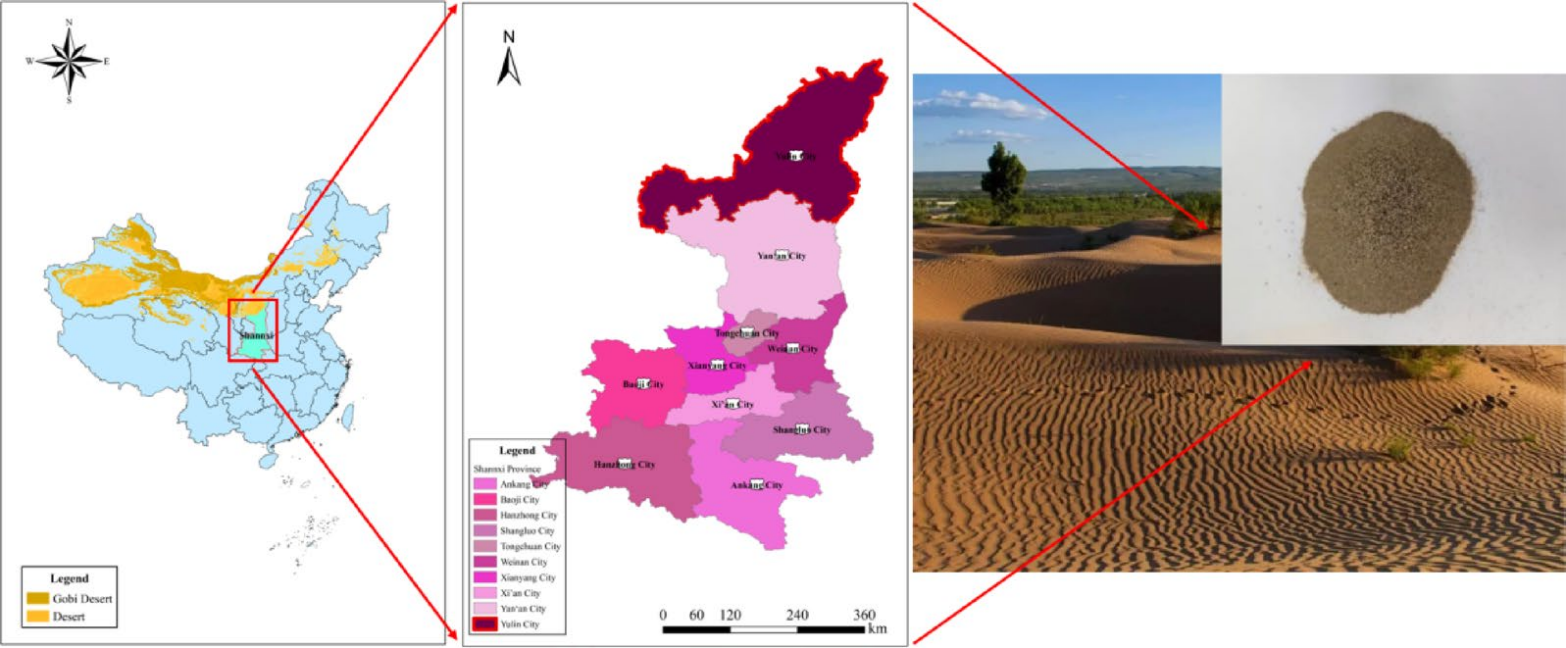
Sample collection point. Maps were generated using ArcGIS Desktop 10.8 (Version 10.7.0.10450) (https://my.esri.com).

**Fig. 2:**
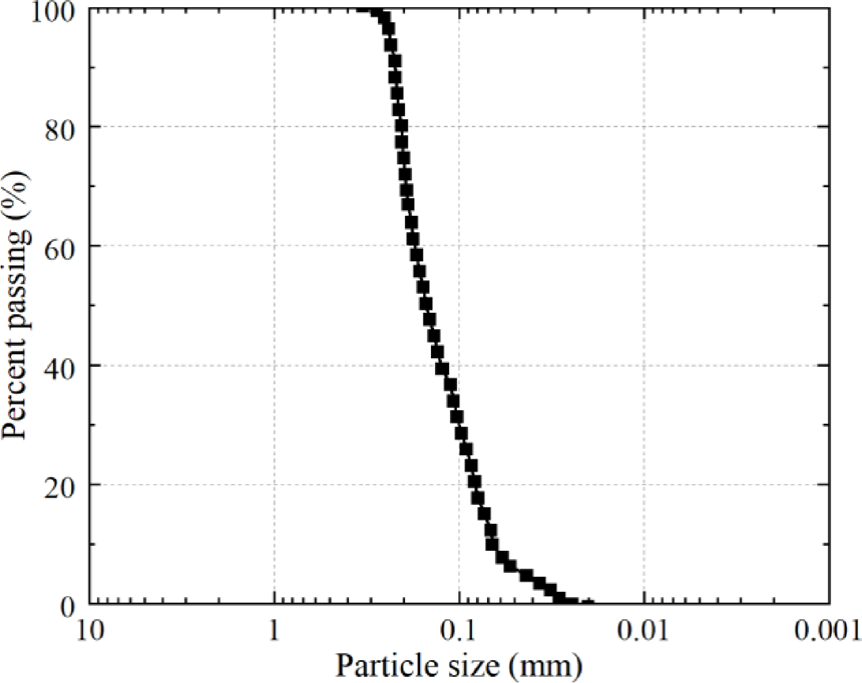
Grading curve of aeolian sand.

The aeolian sand utilized in this experiment was sourced from the Maowusu Desert, located in Yulin City, Shaanxi Province. As shown in Fig. 1, the sand is yellow, free of impurities, with a uniform texture. By the standard for geotechnical test methods (GB/T50123-2019)^28^, the basic physical properties of aeolian sand were tested, as shown in Table 1. Based on the curvature coefficient (C_c_) and non-uniformity coefficient (C_u_), it was determined that the standard sand is poorly graded. The particle size distribution curve is shown in Fig. 2.

**Table 1:** Basic physical properties of the aeolian sand.

G_s_	ρ (g/cm^3^)	w (%)	e	w_*p*_ (%)	w_L_ (%)	I_*p*_	C_u_	C_c_
2.65	1.59	2.60	0.67	19.6	25.0	5.4	2.30	0.85

Basalt fiber (BF) and wool fiber (WF) were purchased from the market. BF is a novel, inorganic, environment-friendly, green, and high-performance fiber material with a smooth surface in brown or bronze. It has various outstanding properties, including high strength, corrosion resistance, and high temperature resistance. WF is a natural protein fiber with a milky white color, mostly clustered together, and has good softness, elasticity, and wear resistance. The urease solution is derived from the supernatant after centrifuging the soybean liquid. The soybeans produced in Suihua City, Heilongjiang Province, are dried and crushed to obtain soybean powder. After screening, deionized water is added to prepare a mixture with a density of 100 g/L, and a magnetic stirrer is used to stir it into the soybean liquid. The curing solution consists of calcium chloride solution and urea solution. The calcium source of EICP is provided by anhydrous calcium chloride, which has a relative molecular mass of 110.99, appears as white cubic crystals, and is easily soluble in water. Urea has a relative molecular mass of 60.06, appears as transparent rod-shaped crystals, and is also readily soluble in water. According to the continuous attempts of our research group, using the method of Specifications and Procedures for Reagents and Standard-Grade Reference Materials^29^anhydrous calcium chloride and urea were uniformly mixed with deionized water to prepare a solution with a concentration of 1.25 mol/L.

### Sample preparation

**Fig. 3:**
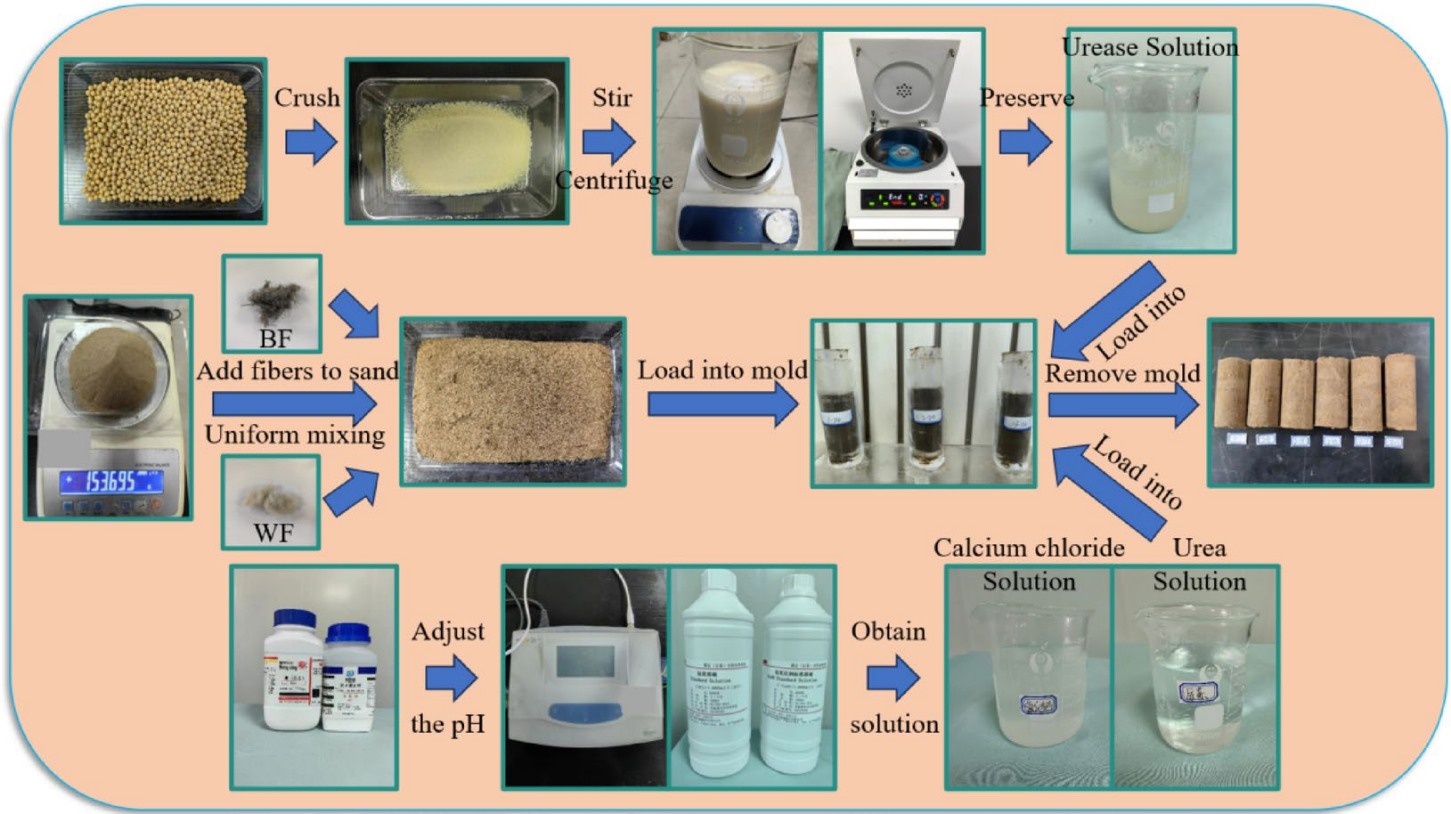
Chart of preparation procedure for sample.

The sand was sieved from a 0.5 mm sieve and subsequently placed in an oven set at 120 °C for 24 h of baking. Set it aside for later use. The PVC pipe, serving as the sample mold, has an internal diameter of 39.1 mm and stands at a height of 150 mm. The sample measures 39.1 mm in diameter and 80 mm in height. For EICP solidified aeolian sand samples, according to the standard for geotechnical test methods (GB/T 50123 − 2019)^28^, different masses of aeolian sand were weighed according to the dry density set in the test. The weighed aeolian sand was divided into four layers and loaded into the mold, and the layers were scraped between them. Before loading the sample, two layers of filter paper were placed at the bottom, and a layer of filter paper was placed on top of the sample. After the sample preparation was completed, a two-stage method was used to inject urease solution and curing solution into the sample. In the first stage, urease solution was injected, and in the second stage, calcium chloride solution and urea solution were injected sequentially. After 24 h, the next round of infusion was carried out. After completing all infusion cycles and reaching the settling time, rinse the sample with deionized water three times to terminate the internal reaction. After that, the mold was detached, and the sample was put in an oven at 80 °C for 36 h to dry. Among them, the injection amounts of calcium chloride solution and urea solution remained constant at 15 mL each, while the injection amount of urease solution varied with the change of enzyme cement ratio. For EICP collaborative fiber reinforced samples, different masses of aeolian sand were weighed according to the dry density set in the experiment. A certain length and content of fibers are disassembled using a dry mixing method, evenly mixed into the aeolian sand, and the mixed aeolian sand was divided into four layers and loaded into the mold, with scraping treatment between layers. The subsequent steps are the same as above. The specific preparation procedure for a sample is shown in Fig. 3.

### Test method

In order to obtain the optimal solidification conditions for EICP solidification of aeolian sand, permeability tests and unconfined compressive strength tests were conducted according to the standard for geotechnical test methods (GB/T 50123 − 2019)^28^. The influence factors, such as dry density (*ρ*_d_), bonding times (*n*), standing time (*t*), and enzyme cement ratio (*a*), were considered during the test process. The test scheme is shown in Table 2. The penetration test was conducted using a self-made permeameter, with a total of 81 groups and repeated tests set for each group. During the experiment, a variable head permeability test method was used to determine the permeability coefficient of EICP solidified aeolian sand. The unconfined compressive strength test was conducted using the KTL-LDF50 soil static triaxial testing machine produced by Xi’an Kangtuoli Instrument Co., Ltd. (Xi’an, China), with a total of 81 sets and repeated tests for each set. During the experiment, the entire process was conducted at room temperature (25$\:\pm\:$1°C) and natural humidity. The confining pressure of the testing machine is set to 0 kPa, and vertical pressure is uniformly applied at an axial strain rate of 1%/min. At the same time, the pressure value displayed on the testing instrument and the deformation of the specimen are observed and recorded. Among them, the bonding times refer to the number of cycles of solution infusion during the EICP curing process. Standing time refers to the total interval time after each perfusion of the sample is completed. The enzyme cement ratio refers to the volume ratio of the injected urease solution to the curing solution (calcium chloride and urea solutions). The control of the enzyme cement ratio is to maintain the volume of the added curing solution constant and change the volume of the added urease solution. The generated amount of CaCO_3_ is the quality difference between before and after the sample was solidified.

**Table 2:** Test scheme of EICP solidified aeolian sand.

ρ_d_ (g/cm^3^)	*n*	t (d)	a
1.40	1	1	1:1
1.50	3	3	2:3
1.60	5	5	1:2

Based on the optimal curing conditions, unconfined compressive strength tests were conducted to obtain the optimal fiber reinforcement conditions by EICP combined with fiber reinforcement technology. The influence factors, such as fiber type (*FT*), fiber length (*FL*), and fiber content (*FC*), were considered during the test process. The peak strength obtained by EICP combined with fiber reinforcement technology was compared with the peak strength obtained by EICP technology under the optimal conditions, and the changes in the strength improvement rate (*q*) were analyzed. The test scheme is shown in Table 3. A total of 32 groups of tests were conducted, with each group configured for repetitive testing. The mechanism of EICP combined with fiber reinforcement in solidifying aeolian sand was analyzed. Ultimately, based on the test results of UCS, a Poly model considering the effects of f *FL* and *FC* was established and verified.

**Table 3:** Test scheme of EICP combined with fiber reinforcement solidified aeolian sand.

FT	FL (mm)	FC (%)
BF	3	0.25
	6	0.50
	9	0.75
	12	1.00
WF	3	0.25
	6	0.50
	9	0.75
	12	1.00

## Results and analysis

### Analysis of the results of penetration test by EICP solidified aeolian sand

**Fig. 4:**
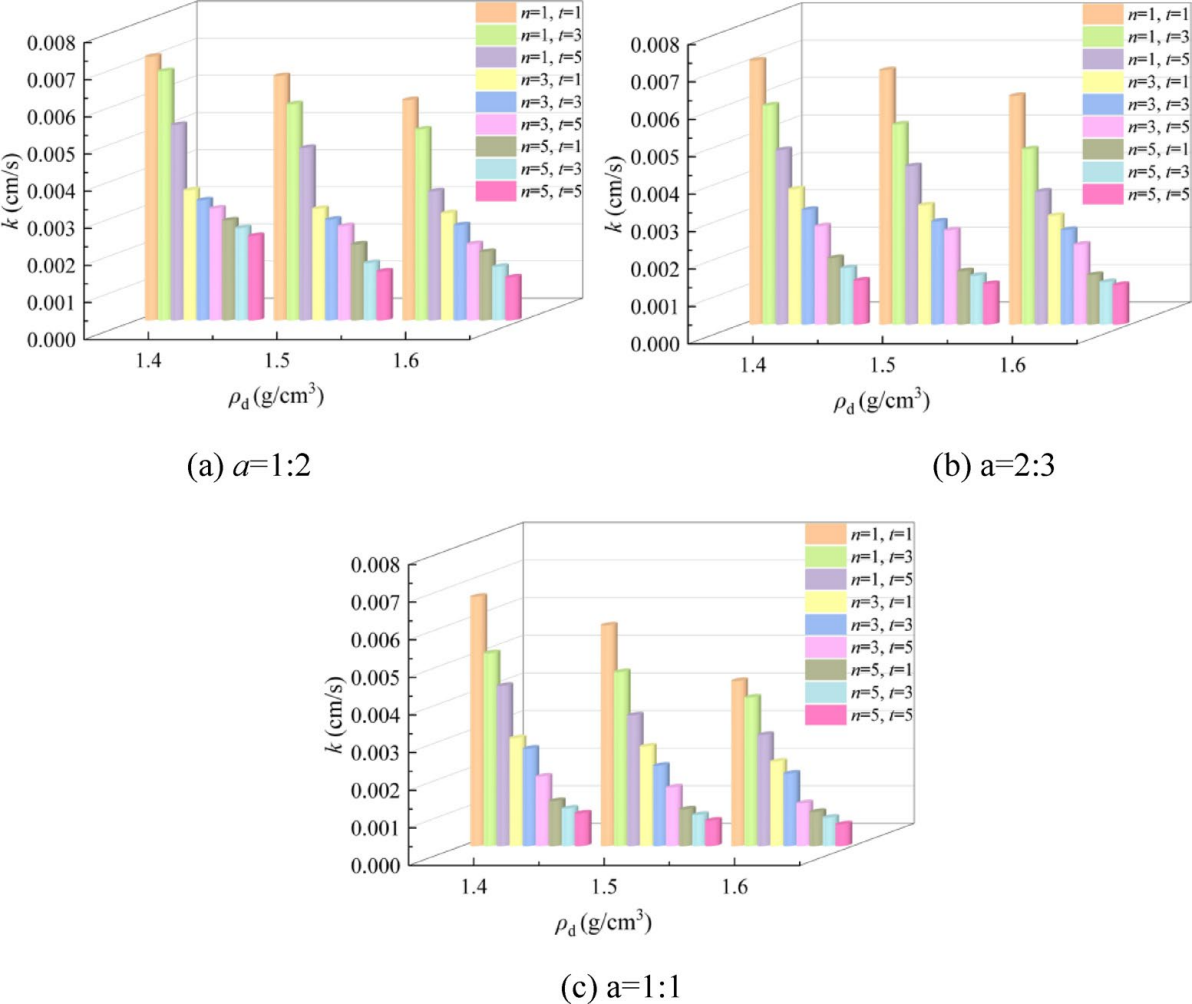
Bar chart between k and different influencing factors.

The size of the permeability coefficient (*k*) can reflect the effectiveness of EICP in solidifying aeolian sand^30^. Figure 4 shows the bar chart of *k* and different influencing factors by the EICP solidified aeolian sand. It can be observed from the figure that the *k* decreases continuously with the increase of *ρ*_d_, *n*, *t*, and *a*, reducing from 7.1 × 10^− 3^ cm/s to 5.8 × 10^− 4^ cm/s, which is reduced by an order of magnitude. The primary cause for the above situation is that the products of the EICP reaction fill up the pores between the sand particles, effectively reducing the *k* of the solidified sand, which is consistent with the research results of Cui et al.^31^. With the increase of *a*, the rate of decline in *k* increases. The primary cause for this situation is that the rise of urease provides sufficient conditions for the EICP reaction. The smaller a is, the more solid solution is added. Excessive Ca^2+^ and urea concentration limit the progress of the EICP reaction^32^because high concentrations of Ca^2+^ will inhibit urease activity and reduce urea decomposition, reducing the yield of CaCO_3_. In addition, as the number of *n* increases, the larger *a* is, the lower the rate of decline of *k* is. The main reason is that the more a, the more CaCO_3_ is generated, and the pores between the sand particles become smaller and smaller, resulting in the infiltration rate of the solution becoming slower until it is less than the reaction rate. Therefore, the generated substances block the infiltration port, resulting in a decrease of the infiltration path.

### Analysis of the results of the UCS test by EICP solidified aeolian sand

**Fig. 5:**
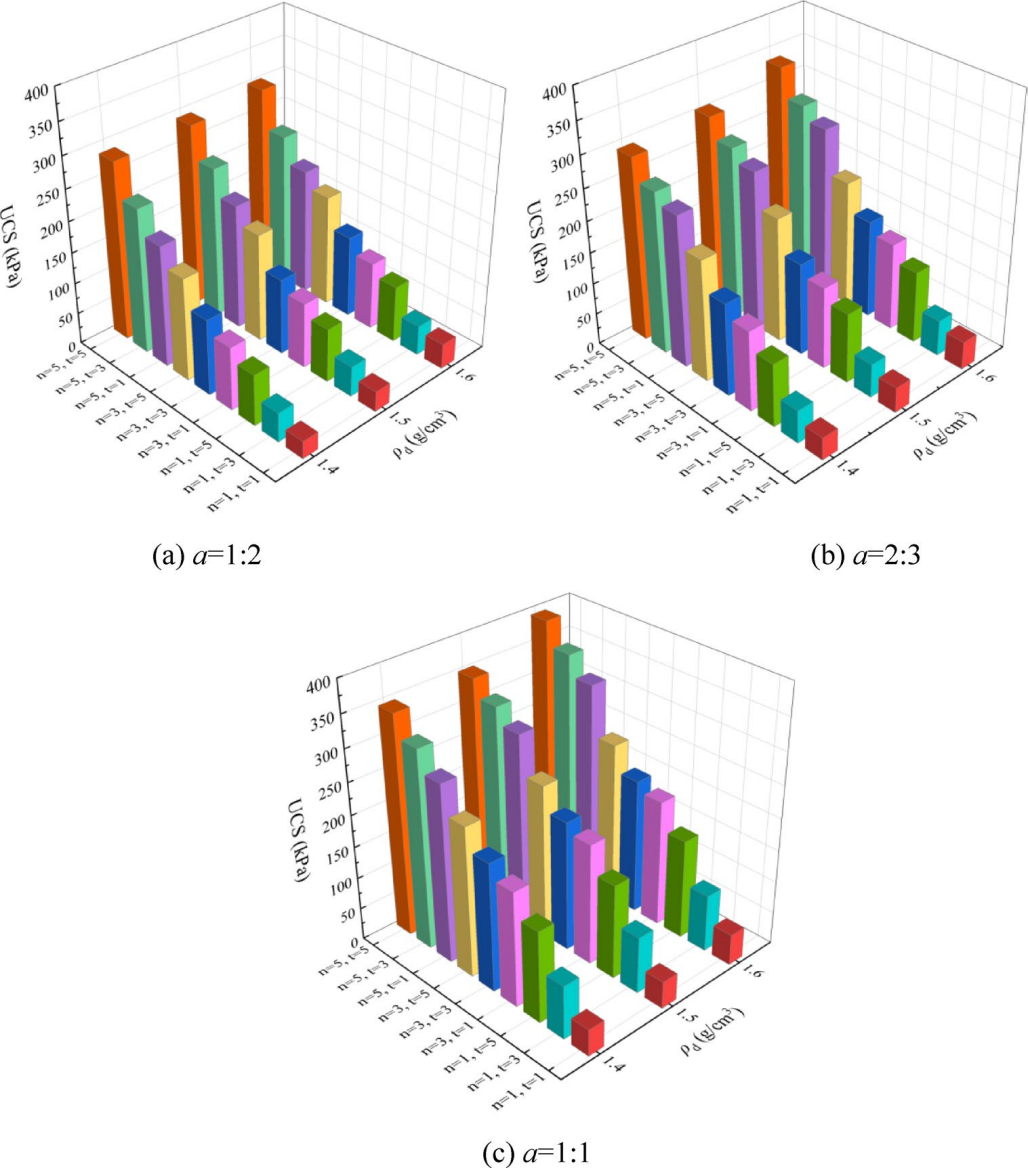
Bar chart between UCS and different influencing factors.

UCS is an important indicator of soil mechanical properties, which can be used with *k* of the sample to verify the curing effect of EICP technology. Figure 5 shows the bar chart between UCS and different influencing factors by the EICP solidified aeolian sand. It can be observed from the figure that the UCS increases with the increase of *ρ*_d_, *n*, *t*, and *a*, and is inversely proportional to *k*. When the *ρ*_d_, *n*, *t*, and *a* are 1.6 g/cm^3^, 5 times, 5 days, and 1:1, the UCS of the sample reaches a maximum of 392.52 kPa, and the k reaches a minimum of 5.8 × 10^− 4^ cm/s. As the EICP reaction continues, the generation of CaCO_3_ makes the internal pores of the sample smaller and smaller, and the UCS gradually rises with the continuous increase in cementation strength, which is consistent with the research results of Zhang et al.^33^. Under the action of uniaxial load, the sample’s longitudinal strain gradually increases, and the internal sand particles move horizontally due to the Poisson effect^34^ and become dense. As the load increases, the sample undergoes brittle damage without significant deformation. The slits expand from the upper and lower ends to the middle. The surface sand particles slowly fall off as the cracks extend, and the sample eventually is destroyed. This phenomenon is more apparent when the aeolian sand’s *ρ*_d_, *n*, *t*, and *a* increase. The primary cause for this phenomenon is that the greater the *ρ*_d_, the smaller the distance and pores between the sand particles, and the more significant the filling effect of the products generated by the EICP reaction. The more *n* is, the more CaCO_3_ is generated, which can more effectively wrap and cement the sand particles. The longer *t* means the EICP reaction takes longer, the more complete the reaction will be. The larger the *a* is, the higher the urease content, the Ca^2+^ and urea concentration are relatively lower, and the conditions required for the EICP reaction are more favorable, which is consistent with the research results of Zhang et al.^35^.

### Analysis of the relationship between *k* and UCS with the yield of CaCO_3_

**Fig. 6:**
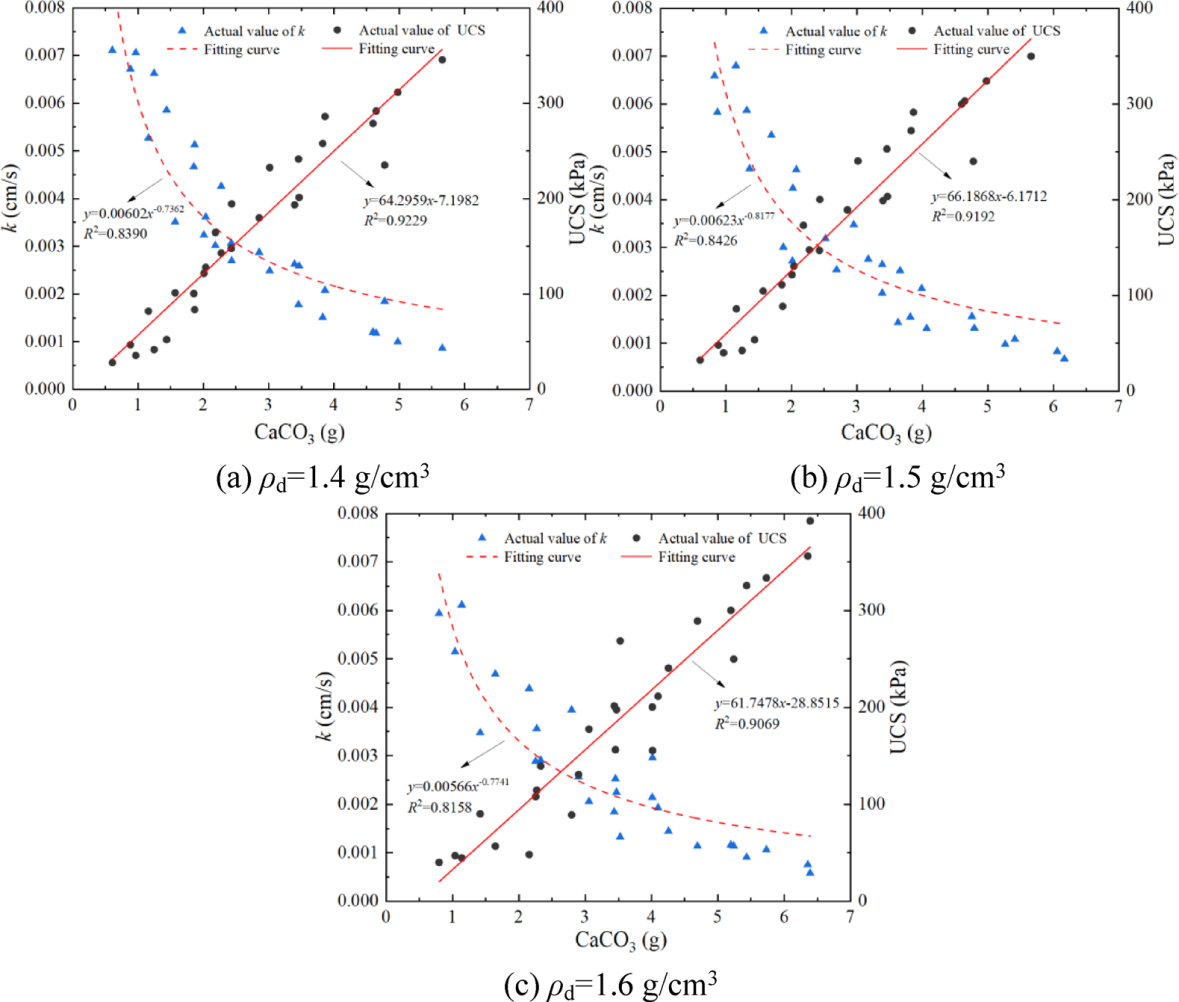
Fitting curve between the *k*, UCS and the yield of CaCO_3_.

The content and distribution of CaCO_3_ directly determine the strength of the sand. Figure 6 shows the fitting curve between the *k*, UCS, and CaCO_3_ generated by the EICP solidified aeolian sand. It can be observed from the figure that for different *ρ*_d_, the more CaCO_3_ is generated, the smaller *k* is, and the larger UCS is. The UCS is exponentially related to the yield of CaCO_3_, which agrees with the result of reference^36^. The determination coefficient of the fitting curve decreases with the increase of *ρ*_d_. The primary cause for this phenomenon is that the larger the dry density, the smaller the pores between the sand particles, and the solidified solution will react during the infiltration process, resulting in more CaCO_3_ being generated at the infiltration port, resulting in uneven solidification of the sample. The smaller the *ρ*_d_, the higher the generation rate of CaCO_3_. This is because the smaller the density, the larger the pores between the sand particles, which provides sufficient space for the EICP reaction. The increase in CaCO_3_ generation means that the internal pores of the sample are reduced; in other words, the seepage path is reduced, so the *k* is negatively correlated with the CaCO_3_ generation. The more CaCO_3_ is generated, the better the sample bonding effect, and the mechanical properties are enhanced, so the UCS is positively correlated with the CaCO_3_ generation. The optimal conditions of EICP solidified aeolian sand obtained by permeability test, unconfined compressive strength test, and combined with CaCO_3_ generation are that *ρ*_d_, *n*, *t*, and *a* are 1.6 g/cm^3^, 5 times, 5 days, and 1:1, respectively. This conclusion lays the foundation for subsequent experiments.

### Analysis of the effect of *FT* on the solidification of aeolian sand

**Fig. 7:**
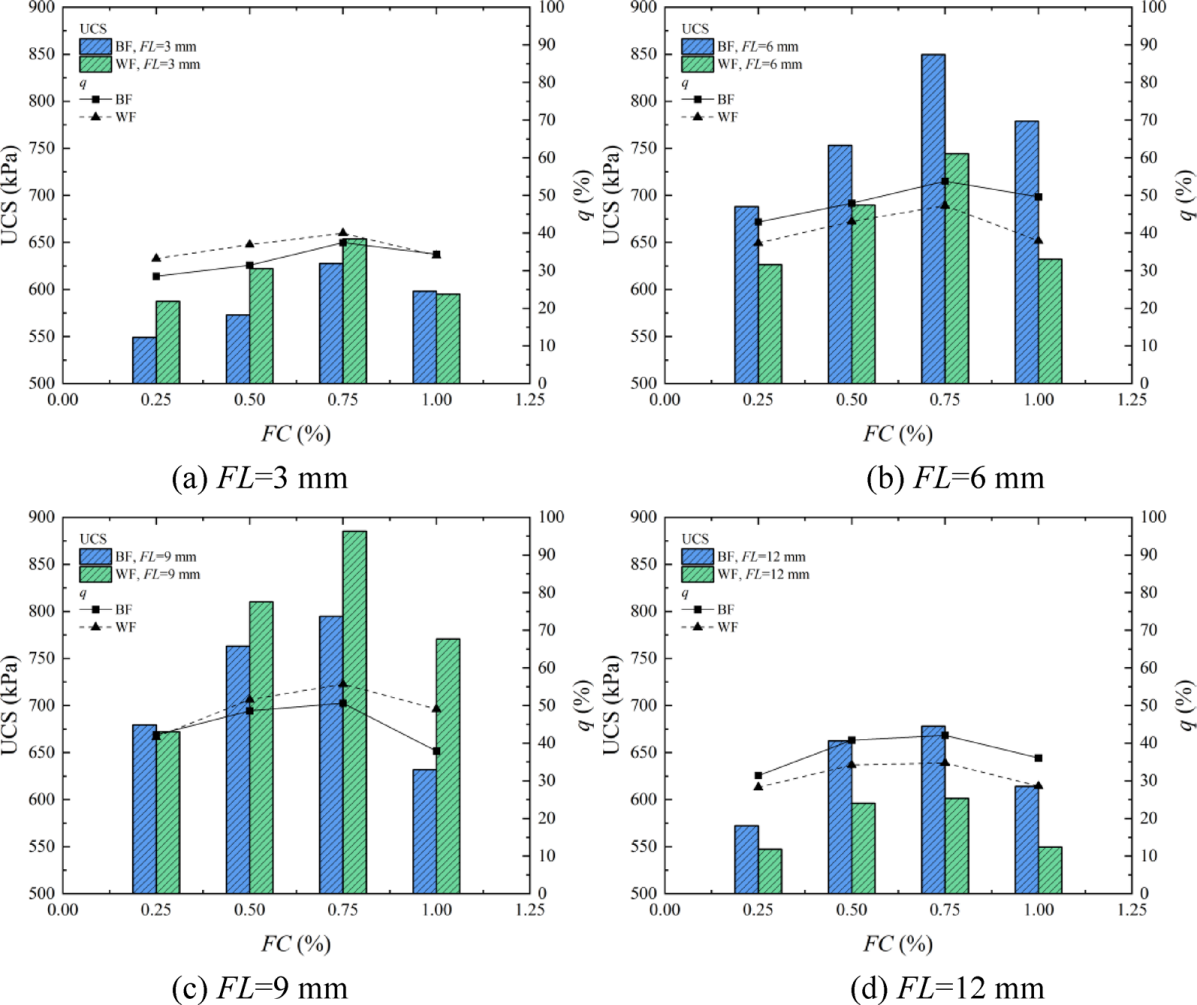
Bar chart between UCS and the type of fiber.

One of the main functions of fiber-reinforced aeolian sand is to enhance soil strength. The addition of fibers changes the original stress path of the sand, improving the overall strength of the sand^37^. Figure 7 shows the relationship bar chart between UCS and fiber type by EICP, combined with fiber reinforcement solidified aeolian sand. The *q* is the peak strength improvement rate (392.52 kPa) obtained under the optimal conditions after adding fibers. It can be seen from the figure that the strength of the sample is significantly improved after adding fibers. When BF is added, the strength of the sample increases by 28.51–53.80%. When *FL* and *FC* are 6 mm and 0.75%, the maximum strength of the sample reaches 849.65 kPa. When WF is added, the strength of the sample increases by 28.28–55.66%. When *FL* and *FC* are 9 mm and 0.75%, the maximum strength of the sample reaches 885.31 kPa. Comparing the two types of fibers, in most cases, BF reinforcement is better than WF. The reason for this phenomenon is that the diameter of BF is smaller than that of WF, which is more conducive to interpenetration between sand particles and connecting with sand particles. Under the same conditions, the tensile strength of BF itself is better than that of WF^38^. However, as shown in Fig. 5c, when *FL* is 9 mm, the effect of WF reinforcement is significantly enhanced, and the peak strength of the sample is greater than that of BF reinforcement. This is because the rough surface of WF provides additional nucleation sites for the CaCO_3_ formation during the EICP reaction, which can promote the more directional formation of CaCO_3_ in the pores of sand particles, thereby improving the overall strength and toughness of the sample. This is consistent with the research results of Wang et al.^39^so the final peak solidification strength obtained is higher than that of the BF reinforcement.

### Analysis of the effect of *FL* on the solidification of aeolian sand

**Fig. 8:**
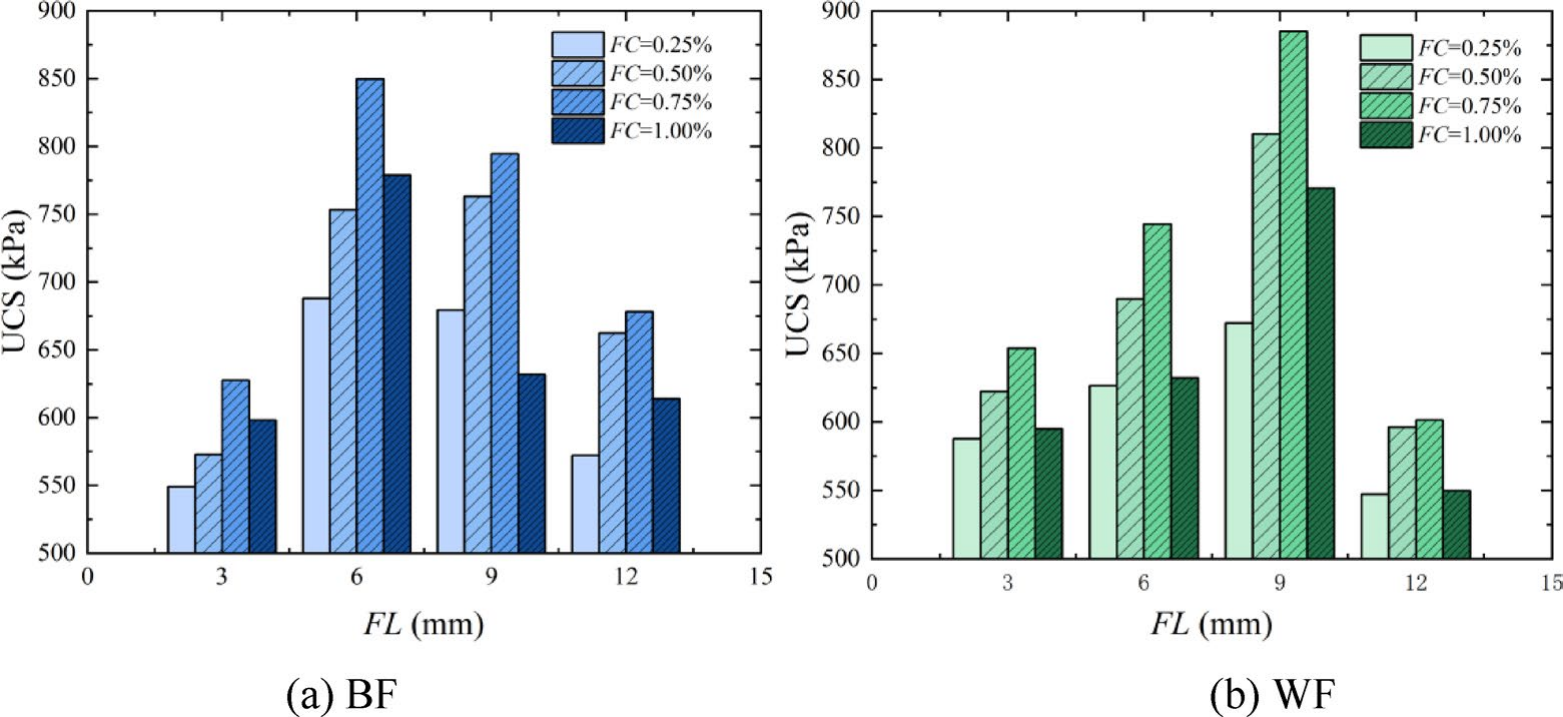
Bar chart of the UCS with the variation of *FL*.

*FL* is one of the important factors affecting the fiber reinforcement effect. Figure 8 shows the bar chart of the UCS with the variation of FL by EICP combined with fiber reinforcement technology. It can be seen from the figure that when the length of BF is 3 –6 mm, the sample’s strength increases with the increase of *FL*. When the length of BF is 9 –12 mm, the sample’s strength decreases with the rise of *FL*. When the peak strength is reached, the optimal reinforcement condition for BF is that *FL* is 6 mm. When the length of WF is 3 –9 mm, the sample’s strength increases with the increase of *FL*. When the length of the WF is 12 mm, the sample’s strength decreases. When the peak strength is reached, the optimal reinforcement condition for WF is that *FL* is 9 mm. The primary cause for this phenomenon is that when *FL* is 3 mm, the fiber is too short and cannot effectively inhibit the expansion of cracks when the sample is damaged^40^. When the *FL* reaches 6 mm, for BF, a shorter fiber is more conducive to filling into the pores of sand particles; it has tensile and compressive resistance better than WF, which improves the sample’s strength. However, for WF, the shorter fiber can result in a small contact surface between the fiber and sand particles, and the three-dimensional network structure formed by the *FL*, sand particles, and CaCO_3_ is not complete, so the fiber cannot resist the evolution of fissures in the sample during the stress process. As the *FL* increases until it reaches 9 mm, the contact surface increases between the WF and the sand particles, and then the friction generated by the mutual contact also increases. When the sand particles move under pressure, the fiber will provide a pulling force under the action of friction, which means that the fiber can absorb more energy at this time, thereby reducing the degree of sample damage and improving compressive strength, consistent with the research results of Dai et al.^41^. However, when the *FL* reaches 12 mm, whether BF or WF, during the stress process of the specimen, the fiber will become knotted or folded inside, forming a weak surface of aggregation. At this time, the force transmission effect of the fiber is inhibited to a certain extent, reducing the UCS of the sample.

### Analysis of the effect of *FC* on the solidification of aeolian sand

**Fig. 9:**
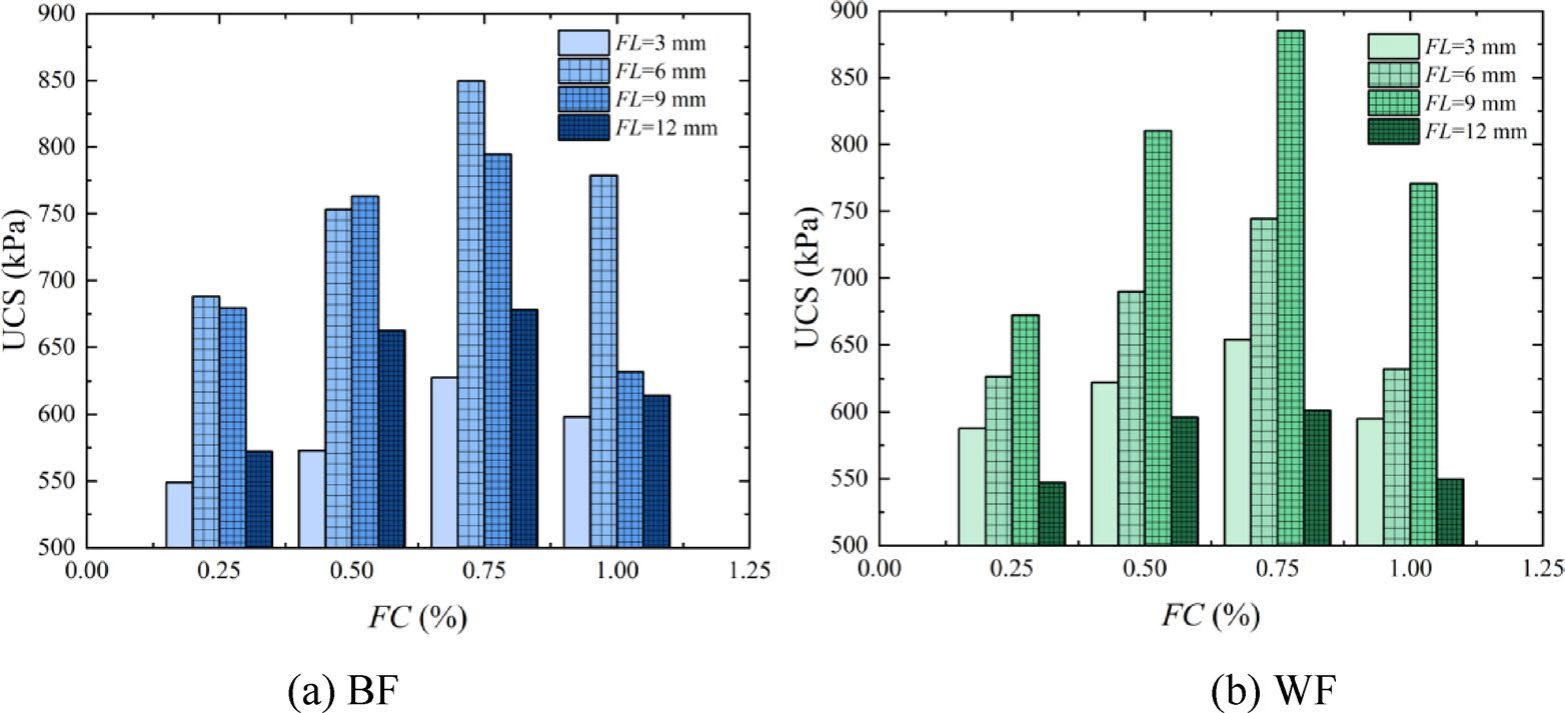
Bar chart of the UCS with the variation of *FC*.

The addition of fibers hinders or delays sand deformation through its excellent mechanical properties, and the *FC* has an essential impact on the fiber reinforcement effect. Figure 9 shows the bar chart of the UCS with the variation of fiber content by EICP combined with fiber reinforcement technology. It can be seen from the figure that when *FC* is 0.25% ~ 0.75%, the sample’s strength exhibits an ascending trend as the *FC* rises. When *FC* is 1.00%, the strength of the sample decreases. Comparing the two fibers, at the peak strength, the optimal reinforcement condition of BF is that *FC* is 0.75%, and the UCS is 849.65 kPa. At the same time, the optimal reinforcement condition of WF is that *FC* is 0.75%, and the UCS is 885.31 kPa. The chief cause for this situation is that when the *FC* is 0.25% ~ 0.50%, the distance between the fibers is elongated, and the network structure cannot be effectively formed to play a “bridging” role. In addition, the fibers are usually subjected to force as a single strand. Owing to the limited toughness of the fibers, when the ultimate tensile strength of a single fiber exceeds its strength limit, the fiber is pulled apart^42^. Therefore, the sample’s strength by EICP combined with fiber reinforcement is higher than that of the sample by EICP solidification. With the increase of *FC*, the contact between the fiber and the sand particles increases, which is more conducive to EICP reaction, providing additional nucleation sites for forming CaCO_3_. As CaCO_3_ content increases, the fibers interweave and overlap with each other in the sand to form a spatial network structure with CaCO_3_ and sand particles. When the sample is deformed due to external force conditions, the spatial network structure bears its pressure, and friction is generated between the fibers, limiting this deformation and increasing strength, which is consistent with the research results of Ren et al.^43^. When *FC* is greater than 0.75%, it is easy for fibers to clump with aeolian sand during the mixing process, which affects the reinforcement effect. In addition, too many fibers occupy a large number of pores between aeolian sand particles, inhibiting the formation of CaCO_3_, causing uneven solidification and resulting in relatively low strength^37^.

### Solidified mechanism of aeolian sand

**Fig. 10:**
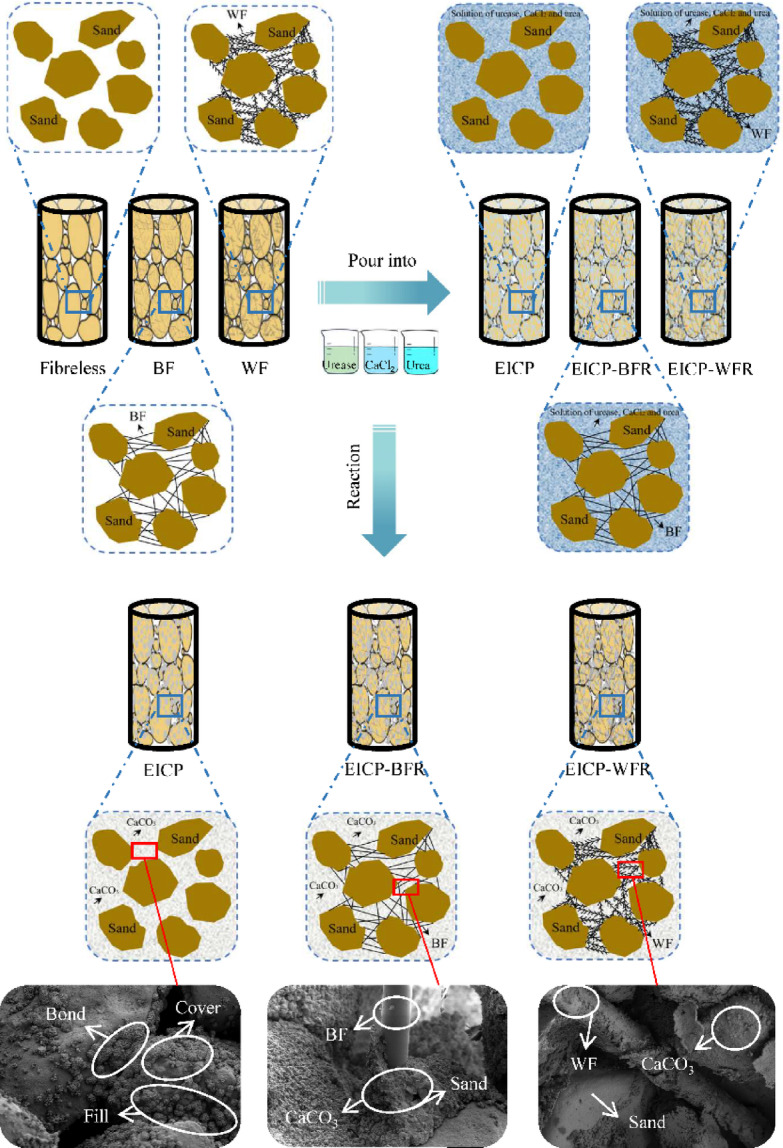
Diagram of the mechanism of EICP combined with fiber reinforcement in solidifying aeolian sand and its enhancement path on mechanical properties. 3D Model was created by Microsoft Office PowerPoint 2021 (Version 2507) (https://1drv.ms/p/c/c83dddabbf0f6889/EbdpjdLZiF1MvzZq8vX2eWMBXKMwGD9ll2Wol4kdVCDLwA?e=3V6Oaz).

Figure 10 shows the mechanism of EICP combined with fiber reinforcement in solidifying aeolian sand and its enhancement path on mechanical properties. It can be seen from the figure that the urease solution first infiltrates into the interior of the sample during the solidification process. The pores and surfaces of the sand particles are filled and covered by urease solution. With the addition of CaCl_2_ solution and urea solution, urease catalyzes the decomposition of urea to generate NH_3_, which increases the local pH and triggers the combination of Ca²⁺ and CO_3_²⁻ to form the cement of CaCO_3_. A bridging structure is formed between the sand particles and fills the pores, achieving a dual effect of physical bonding and chemical solidification. During this process, the sand particles provide nucleation sites for generating and stacking CaCO_3_^44^. The pores between sand particles are relatively large, and the CaCO_3_ generated by EICP mineralization is mainly distributed in the pores and surfaces of sand particles. The addition of fibers creates a three-dimensional reinforced network, with BF mechanically interlocking with CaCO_3_ through surface roughness^45^. At the same time, WF relies on high ductility to generate fracture energy absorption, synergistically limiting sand slip^46^. The color gradient from white to blue and then to gray between the pores of sand particles reflects the solidification process. After EICP combined with fiber reinforcement, the crystallinity of CaCO_3_ and fiber distribution density in sand particles are significantly improved. Through multi-scale reinforcement of nanoscale bonding and millimeter-scale fibers, the UCS, *c*, and *φ* of aeolian sand are synchronously optimized. At the same time, the bridging effect of fibers effectively suppresses crack propagation, ultimately achieving the transformation from brittle sand to a ductile solidified body.

**Fig. 11:**
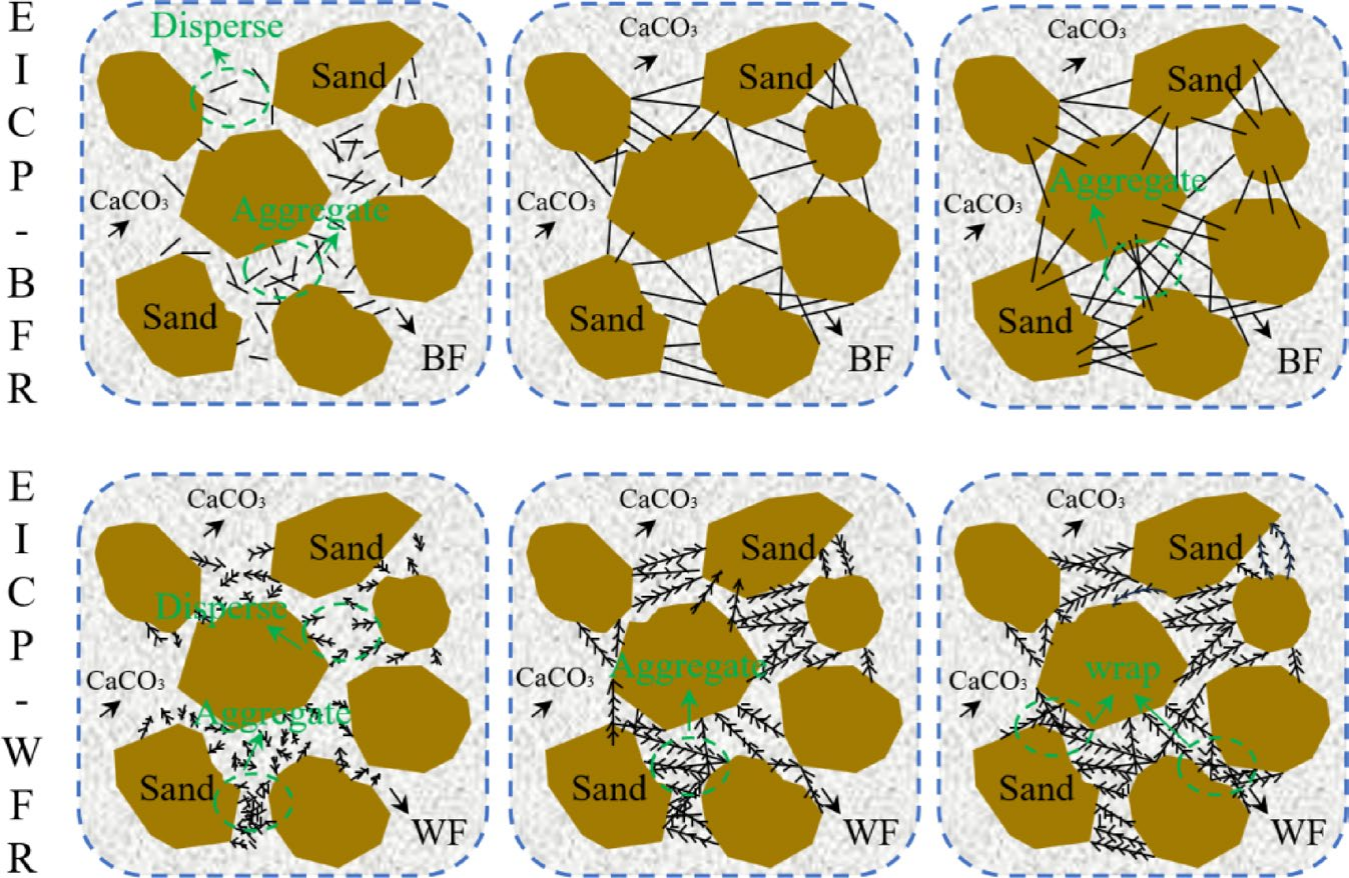
The mechanism of FL and FC on EICP Stabilized aeolian sand. 3D Model was created by Microsoft Office PowerPoint 2021 (Version 2507) (https://1drv.ms/p/c/c83dddabbf0f6889/EbdpjdLZiF1MvzZq8vX2eWMBXKMwGD9ll2Wol4kdVCDLwA?e=3V6Oaz).

Figure 11 shows the mechanism of FL and FC on EICP Stabilized aeolian sand. The enhancement mechanism of *FL* on EICP-stabilized aeolian sand depends on effective stress transfer and interface contact efficiency, and its core theory is the Critical Length^47^. When the *FL* is less than 3 mm, the contact area between the fiber matrix interface of the two fibers is insufficient to effectively bridge cracks, resulting in stress concentration^48^. From Fig. 7, it can be seen that the UCS reaches its maximum when the *FL* of BF and WF are 6 mm and 9 mm, respectively, achieving the optimal length. Due to its high stiffness, BF can penetrate multiple sand pores in a short length, forming a continuous stress skeleton. The WF needs to reach 9 mm to increase the friction interface, activate its high ductility, and absorb fracture energy through fiber stretching. When the *FL* is greater than 12 mm, it is more prone to entanglement and agglomeration during the dry-mixing process, resulting in fiber enrichment in local areas and fiber scarcity in other areas, which leads to uneven strength distribution and overall decline in strength. The enhancement mechanism of *FC* on EICP-stabilized aeolian sand depends on the formation and integrity of the three-dimensional network structure. When the *FC* is less than 0.5%, the fibers are dispersed and isolated, unable to transmit stress, and UCS only relies on CaCO_3_ bonding. When *FC* increases to 0.75%, the fiber spacing decreases, and a spatial grid is formed in coordination with CaCO_3_, blocking the crack propagation path^49^. At this point, the UCS of BF and WF reaches its maximum. WF further optimizes the distribution of CaCO_3_ crystals and enhances interfacial adhesion due to its surface activity. When the *FC* is greater than 0.75%, fiber aggregation occupies too much pore space, which not only hinders the penetration of the solidification solution and limits the effective deposition of CaCO_3_ between sand particles but also makes the aggregates themselves stress concentration points and weak areas, resulting in uneven solidification and decreased strength.

## Establishment and verification of model

### Model establishment

**Fig. 12:**
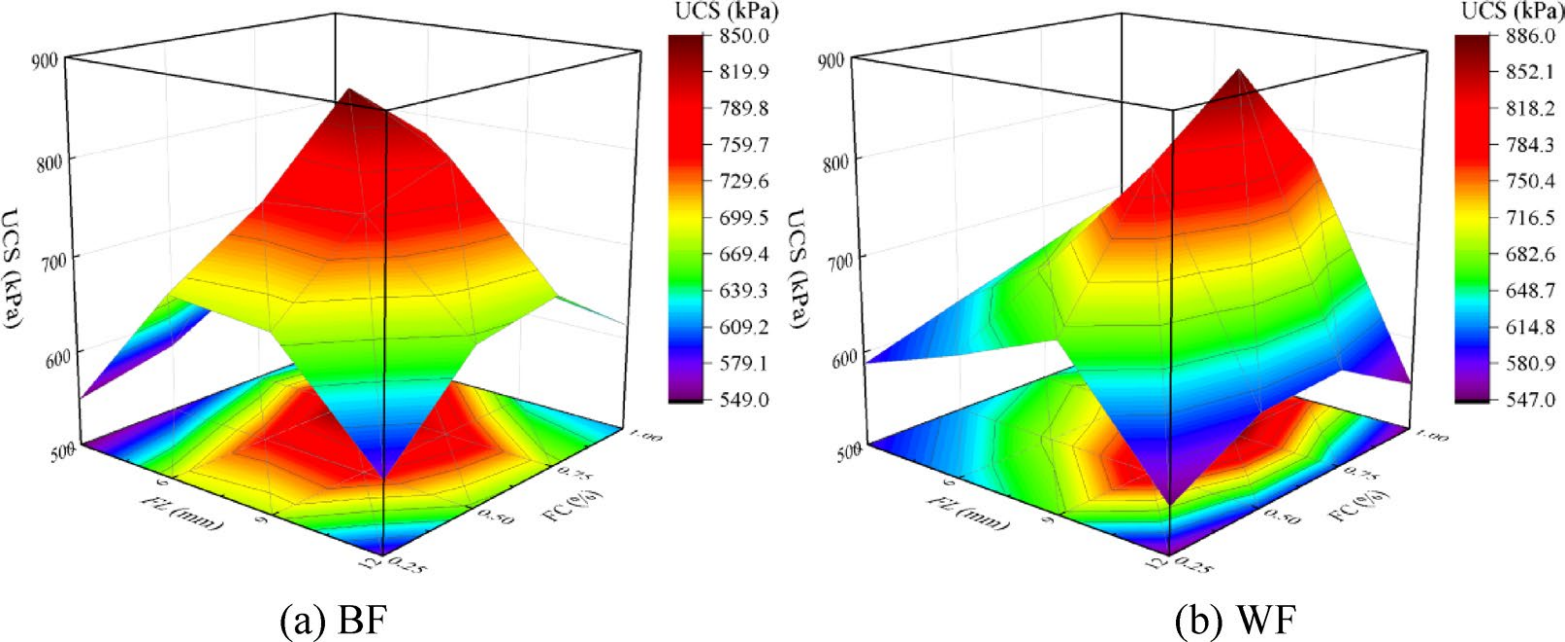
Three-dimensional poly model between *FL*, *FC*, and UCS.

Fiber reinforcement significantly enhances the specimen’s strength and mitigates brittle failure. For different types of fiber reinforcement, under different *FL* and *FC* conditions, the unconfined compressive strength of the solidified aeolian sand initially rises and then falls with increasing *FL* and *FC*. Therefore, a three-dimensional Poly model representing the relationship between *FL*, *FC*, and UCS is established for EICP combined with fiber reinforcement solidified aeolian sand, as shown in Fig. 12. As shown in formula (1), the calculation formula of the Poly model is established, and the X-axis, Y-axis, and Z-axis are sequentially set as the *FL*, *FC*, and UCS, predicting the UCS through *FL* and *FC*.

1 $$U = a\,FL + b\,FC + c\,\left( {FL} \right)^{2} + d\left( {FC} \right)^{2} + f{\text{ }}FL \cdot FC + z$$

where *U* is the unconfined compressive strength (kPa); *FL* is the fiber length (mm); *FC* is the fiber content (%).

### Parameter acquisition

**Fig. 13:**
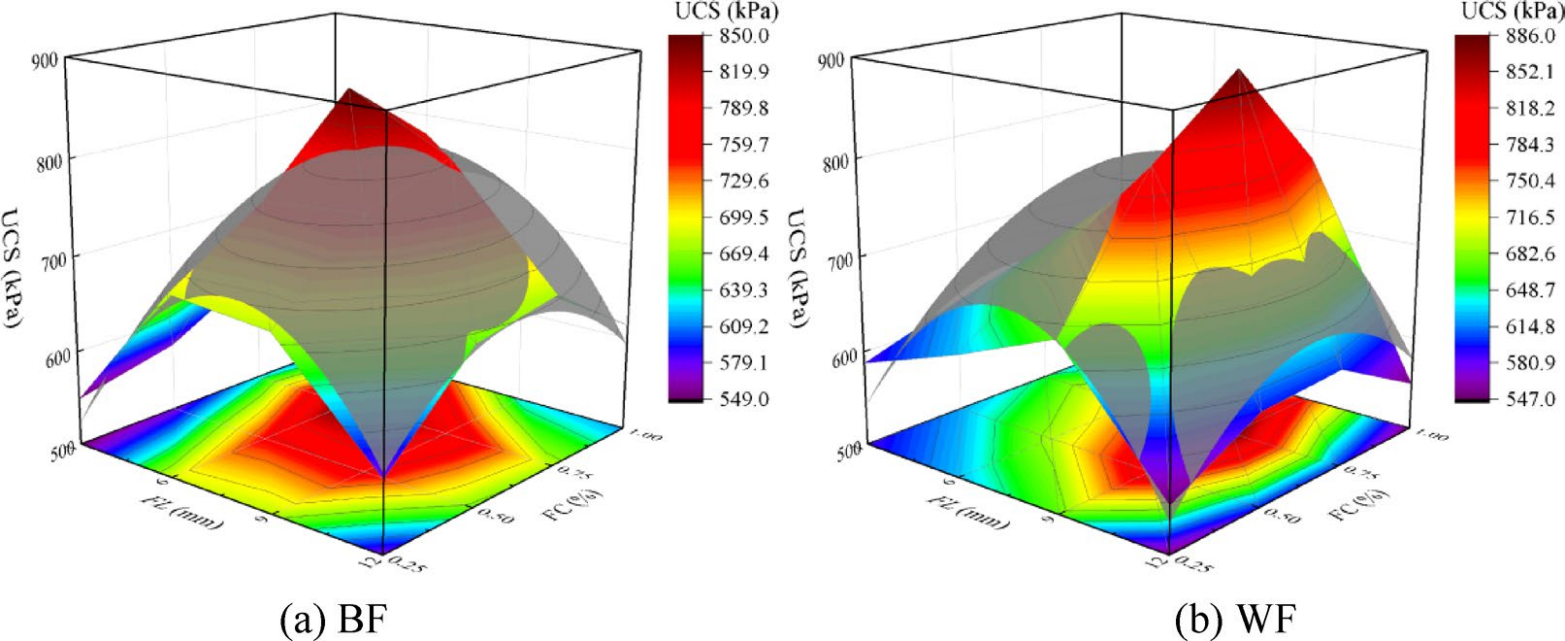
Fitting the curved surface of the poly model.

Figure 13 shows a fitting curved surface of the Poly model by EICP combined with fiber reinforcement technology. It can be seen from the figure that the fitting surface covers the regression analysis surface relatively evenly. In the Poly model of aeolian sand samples solidified by EICP technology mixed with BF and WF reinforcement, the fitting parameters of *a*, *b*, *c*, *d*, *f*, and *z* are shown in Table 4. The R^2^ are 0.9270 and 0.8815, respectively, which indicates that the correlation of *FL*, *FC*, and UCS is strong.

**Table 4:** Fitting parameter of the poly model.

FT	a	b	c	d	f	z	*R* ^2^
BF	119.21	864.35	-7.39	-590.14	-8.88	58.64	0.9270
WF	110.45	810.71	-7.48	-622.01	2.35	112.39	0.8815

### Model verification

**Fig. 14:**
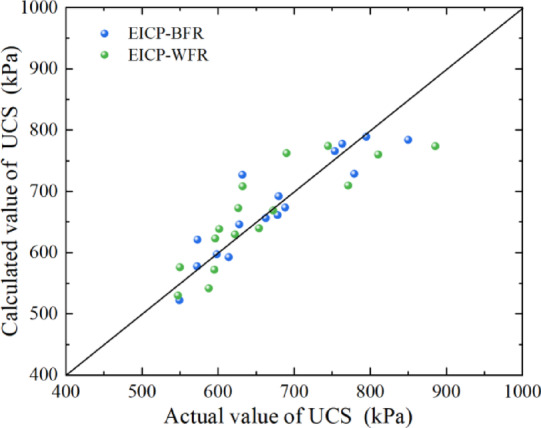
Comparison chart of the measured and calculated values of the UCS.

The measured data and the acquired parameters are substituted into the formula (1), and the UCS is obtained by calculation. Figure 14 shows a comparison chart of the measured and calculated values of the UCS by EICP combined with fiber reinforcement technology. It can be seen from the figure that the data are relatively evenly distributed on either side of the bisector, which indicates that the model’s calculation results are consistent with the experimental results.

In this study, the Poly model (formula (1)) is used to predict the UCS of EICP combined with fiber-solidified aeolian sand, which can intuitively reflect the nonlinear effects of FL and FC on UCS. However, the model is only applicable to specific aeolian sands from the Maowusu Desert (Yulin, Shaanxi) studied in this article and fibers (BF or WF), and the model’s predictions are based on the optimal EICP solidification conditions and specific sample preparation methods determined in this article. In the future, we will conduct a large number of experiments to verify the effectiveness of this model.

## Conclusions

Based on permeability tests and unconfined compressive strength tests of EICP solidified aeolian sand, the optimal conditions for solidifying aeolian sand using EICP were obtained. Based on the optimal solidification conditions, the UCS test of EICP combined with fiber reinforcement solidified aeolian sand was conducted, and the optimal fiber reinforcement conditions were obtained. Finally, a Poly model was established to represent the *FL*, *FC*, and UCS relationship. The main conclusions are as follows:

1. The *k* decreases with the increase of *ρ*_d_, *n*, *t*, and *a*. With the rise of *a*, the decline rate of *k* increases. The larger the *a*, the lower the rate of decrease of *k* with the increase of *n*. The UCS increases with *ρ*_d_, *n*, *t*, and *a* rise. When the *ρ*_d_, *n*, *t*, and *a* are 1.6 g/cm^3^, 5 times, 5 days, and 1:1, respectively, the UCS of the aeolian sand sample reaches a maximum of 392.52 kPa.
2. For different dry densities, the more CaCO_3_ is generated, the smaller k is. On the contrary, the more CaCO_3_ is generated, the larger the UCS is. The optimal conditions for EICP solidified aeolian sand obtained by permeability test, unconfined compressive strength test, and combined with CaCO_3_ generation are that *ρ*_d_, *n*, *t*, and *a* are 1.6 g/cm^3^, 5 times, 5 days, and 1:1, respectively.
3. With the length of BF and WF raised, the sample’s strength initially rises and subsequently declines. The peak strength is the optimal reinforcement length. When the peak strength is reached, the optimal length for BF is that *FL* is 6 mm, and the optimal length for WF is that *FL* is 9 mm. Below the optimal length, fibers are unable to effectively bridge cracks and provide reinforcement. Beyond the optimal length, fibers are prone to entanglement and agglomeration, resulting in uneven distribution of strength and overall decline in strength.
4. When *FC* is 0.25% − 0.75%, the sample’s strength exhibits an ascending trend as the *FC* rises. When *FC* is 1.00%, the strength of the sample decreases. Comparing the two fibers, when reaching the peak strength, the optimal content of BF is that *FC* is 0.75%, and the UCS is 849.65 kPa. At the same time, the optimal content of WF is that FC is 0.75%, and the UCS is 885.31 kPa. Below the optimal content, the fiber spacing is too large, making it difficult to form an effective three-dimensional reinforcement network. Above the optimal content, fiber aggregation hinders solution penetration and uniform precipitation of calcium carbonate, and forms weak areas on its own.
5. Based on the test results of unconfined compressive strength by EICP combined with fiber reinforcement solidified aeolian sand, a three-dimensional Poly model representing the relationship between *FL*, *FC*, and UCS is successfully established. By validating the model, it is found that the calculation results of the model are consistent with the experimental results.

## Data Availability

All data generated or analyzed during this study are included in the published paper. The detailed data could be supplied on demand after corresponding author.
